# Un cas rare d'adénite mésentérique suppurée associée à une invagination intestinale chez un enfant: cas Clinique

**DOI:** 10.11604/pamj.2016.23.148.8590

**Published:** 2016-03-31

**Authors:** Didier Tshibangu Mujinga, François Tshilombo Katombe

**Affiliations:** 1 Faculté de Médecine, Université de Lubumbashi, Cliniques Universitaires de Lubumbashi, Service de Chirurgie, République Démocratique du Congo

**Keywords:** Adénite, invagination, intestinale, occlusion, Adenitis, intussusception, intestinal, obstruction

## Abstract

La panniculite est une manifestation rare au cours des dermatomyosites (DM). L'apparition d'une panniculite au cours d'un traitement par du méthotrexate (MTX) est exceptionnelle et n'a été décrite que dans 3 cas. Nous rapportons l'observation d'une patiente âgée de 50 ans atteinte d'une DM diagnostiquée en 1997 et traitée par une corticothérapie avec une évolution favorable aux plans clinique et biologique. A l'occasion d'une rechute 2 ans plus tard, la corticothérapie a été majorée et du méthotrexate à une dose hebdomadaire de 7,5 mg a été rajouté. L’évolution était rapidement favorable. Dix huit mois plus tard, la patiente présentait de multiples nodules sous cutanés siégeant aux 4 membres et aux fesses, dont l'examen anatomopthologique concluait à une panniculite. Il n'existait aucun signe d’évolutivité de la DM. La dose de prédnisone a été augmentée à 0,5 mg/kg/j toujours en association au MTX mais sans aucune amélioration. Le MTX a été arrêté et les lésions cutanées ont complètement disparu en 2 mois sans aucune récidive avec un recul actuel de 42 mois. Notre observation est particulière par la survenue d'une panniculite chez une patiente ayant une DM traitée par du MTX et illustre la difficulté diagnostique. Cette entité doit être connue malgré son caractère exceptionnel puisque l'arrêt du MTX induit en général la disparition des nodules sous cutanés.

## Abstract (en)

Some cases of suppurative mesenteric adenitis have already been described in the literature but not associated with intussusception. We describe the case of a 3-year-old boy presenting to the department of surgery at the University Hospital of Lubumbashi with bowel obstruction. He was visited elsewhere, in the previous 12 days, for diarrhea, vomiting, fever, coma and treated for cerebral malaria and blackwater fever. Surgery revealed an ileal intussusception and a suppurative mesenteric adenitis whose pyoculture revealed the presence of Enterobacter cloacae, sensitive to norfloxacin. We performed desinvagination, sucked the pus out into a syringe and excized completely the site of suppurative adenitis. The evolution of patient was good. The clinician must know that the association between suppurative mesenteric adenitis and intussusceptions exists. The diagnosis is not easy and there is the risk of developing acute peritonitis due to its fistulation in the abdominal cavity.

## Introduction

L'invagination intestinale aigue est une des urgences abdominales les plus fréquentes du nourrisson. Parmi les causes infectieuses d'invagination intestinale les plus fréquemment citées, il y a le rotavirus et le Yersinia enterocolitica. Ces germes sont à la base des adénites mésentériques. Parmi les causes d'adénite suppurée du mésentère, le staphylocoque et le streptocoque ont été cité, mais nous n'avons pas retrouvé dans la littérature l'Enterobacter cloacae comme cause de suppuration des ganglions mésentériques. Nous décrivons un cas rare d'un enfant de 3 ans et 6 mois ayant présenté une invagination intestinale aigue probablement causée par une adénite mésentérique suppurée dont la culture du pus a révélé la présence d'Enterobacter cloacale. L'adénite mésentérique suppurée à Enterobacter cloacae associée à une invagination intestinale n'a pas encore été décrite dans la littérature. Le diagnostic de l'invagination intestinale aigue est urgent. Le clinicien doit savoir qu'il existe un risque de péritonite au cas où l'adénite mésentérique suppurée arrivait à s'ouvrir dans la cavité péritonéale.

## Patient et observation

**Figure 1:**
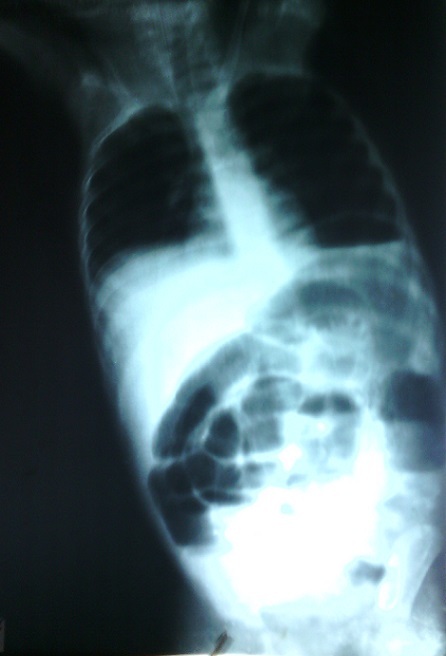
Radiographie de l'abdomen à blanc qui montre des niveaux hydroaériques centraux plus larges que haut et une dilatation importante du grêle rempli de gaz prédominant dans la fosse iliaque gauche, le flanc gauche, l'hypochondre gauche. Une opacité hypogastrique et dans la fosse iliaque droite

Il s'agit d'un garçon de 3 ans et 6 mois pesant 15 kg dont la symptomatologie remonte à 12 jours de l'intervention chirurgicale par des douleurs abdominales, une diarrhée liquidienne, la fièvre et l'anorexie. Il a été soigné en périphérie pendant 7 jours. Il y a reçu deux transfusions et une perfusion de quinine à des doses inconnues. Ceci sans amélioration de son état. Il est alors transféré au service de pédiatrie des Cliniques Universitaires de Lubumbashi avec le tableau clinique suivant: fièvre, obnubilation, urine d'aspect coca cola, sécheresse buccale, pas raideur de nuque, le pli cutané abdominal paresseux et sans plainte en rapport avec la sphère ORL. Des examens paracliniques dans le sang ont révélé: Hb 10,6g, Hte 33%, Groupe Sanguin O, Rhésus positif, glycémie 155mg. La goutte épaisse n'a pas été faite. Il a été traité pour neuropaludisme et fièvre bilieuse hémoglobinurique. Ce traitement était fait de 3 perfusions (sérum glucosé 5% 10ml/kg plus la quinine 10mg/kg) qui coulent chacune pendant 4 heures avec des intervalles de 4 heures entre elles. Une réhydratation faite d'un mélange de sérum glucosé 1L et Sérum physiologique 500ml lui est administré. Au premier jour d'hospitalisation il a émis des mélénas, présenté des convulsions tonico-cloniques (2 fois). Il était pâle et en coma stade II. Il a reçu comme traitement: diazépam 7mg en IM, Duphalac 1 sachet 3 fois par jour par la sonde nasogastrique. Une ponction lombaire a été réalisée mais l'analyse du liquide céphalorachidien n'a révélé aucune anomalie. Un cooling (siphonage de l'estomac avec du sérum physiologique ou de l'eau froide) a ramené du sans noirâtre. Au deuxième jour le patient était lucide, sans mélénas et sans convulsion. Le gavage par sonde nasogastrique était poursuivi. Au troisième jour il a présenté des vomissements alimentaires puis bilieux 5 fois, des douleurs abdominales, la soif et la non émission des selles et des gaz. Il avait des cernes oculaires, un ballonnement abdominale et le pli cutané abdominale était paresseux. Les examens de laboratoires ont montré: Hb 8,6g et glycémie 62mg/dl. Il a été réhydraté et a reçu Pedifen 10ml deux fois par jour. Au quatrième jour les signes de la veille se sont aggravés et Il a reçu le traitement suivant: perfusion de sérum physiologique 400ml + sérum glucosé 5% 600ml + KCl 45mEq pendant 6 heures et une canule rectale a été placée. La radiographie de l'abdomen sans préparation incidence face en position debout avait révélé la présence des niveaux hydroaériques plus larges que hauts prédominant au centre, une opacité dans l'hypogastre et la fosse iliaque droite (Figure 1). Ce qui a motivé son transfert en chirurgie. En chirurgie les plaintes sont les mêmes que celles présentées en pédiatrie. Les signes vitaux montrent une tachycardie à 123bpm, une fréquence respiratoire à 30 cycles par minute et la température à 36,8°C. Le patient est léthargique, avec cerne oculaire, pâleur des conjonctives, sécheresse buccale et une sonde nasogastrique qui ramène un liquide fécaloïde.

**Figure 2:**
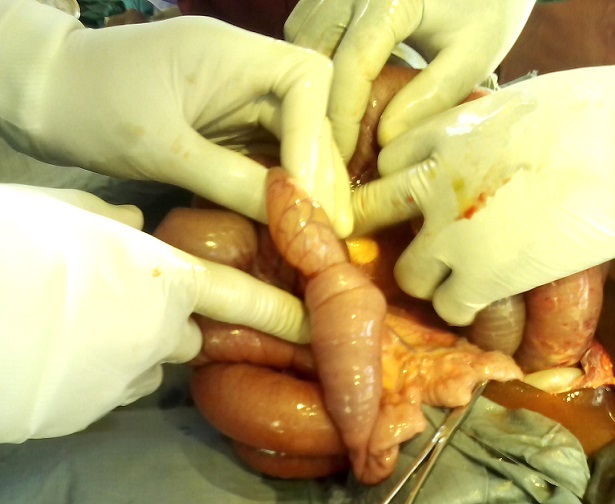
Boudin d'invagination

**Figure 3:**
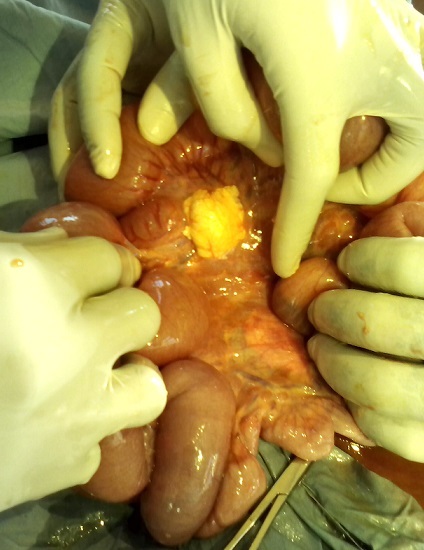
La désinvagination est réalisée: on voit que l'intestin est sain mais sur le mésentère plusieurs ganglions hypertrophiés et une masse jaune (adénite mésentérique suppurée) au sein de ces ganglions

**Figure 4:**
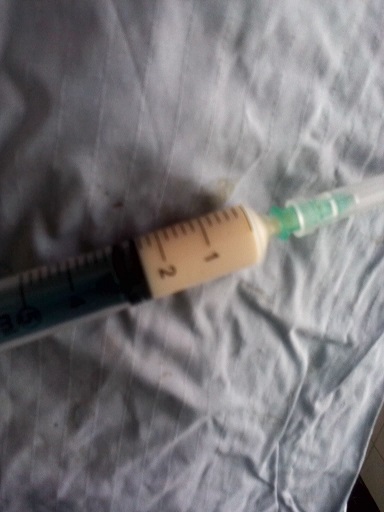
La seringue qui contient le pus aspiré par ponction de l'adénite mésentérique suppurée

L'abdomen entièrement ballonné, les ondes péristaltiques de lutte spontanément visibles sous la peau et exacerbées par la chiquenaude et plus marquées en sus ombilicale. L'abdomen était souple, tympanique dans son ensemble et des bruits hydroaériques en sus ombilical et paraombilcal gauche et un silence dans la région hypogastrique et la fosse iliaque droite. Le boudin d'invagination n'a pas été palpé. Au toucher rectale l'ampoule rectale était vide, le Douglas non bombant, non sensible, pas de masse pelvienne palpée et le doigtier propre. La diurèse à son admission en chirurgie n’était pas connue. Le diagnostic différentiel était une occlusion intestinale haute sur paquet d'ascaris ou sur invagination intestinale aigue. Nous avons fait couler 750ml de sérum physiologique en 1heure et recueilli 120ml d'urine. Le bilan préopératoire avait donné ce qui suit: Hb 8,7g, Hte 26%, GS O, Rh +, TS 1'00", TC 4'30", GB 14050/mm^3^, VS 48mm/h, Formule leucocytaire: N 59%, L 41%. Une intervention chirurgicale a été réalisée dont voici le protocole: Laparotomie médiane sus et sous ombilicale de 12cm de long. Le constat: dilatation importante du grêle depuis l'angle de Treitz jusqu’à environ 50cm de la jonction iléocoecale, le boudin d'invagination iléoiléale de 10 cm de long à environ 50cm de l'angle iléocoecale (Figure 2), le grêle en aval du boudin d'invagination est aplati. Sur le mésentère il y avait plus de 5 ganglions mésentériques hypertrophiés d'environ 2cm de diamètre, immobiles (Figure 3); parmi ces ganglions mésentériques hypertrophiés il avait une masse de couleur jaune (Figure 3), fluctuante d'environ 3cm de diamètre dont la ponction exploratrice a ramené 2,5cc d'un pus jaune (Figure 4) mal lié homogène qui a été envoyé à la bactériologie. Cette masse jaunâtre ne communiquait pas avec la lumière intestinale. Apres désinvagination, l'anse invaginée a gardé une coloration normale (Figure 3). On a incisé la paroi de cette masse et exciser la membrane pyogène dans son entièreté. On a nettoyé la cavité abdominale avec 1l de sérum physiologique tiède puis assécher la cavité abdominale. La paroi abdominale a été fermée sans drainer la cavité abdominale. La membrane pyogène excisée n'a pas été envoyé à l'anatomopathologie. Le patient a subit une réanimation post opératoire, et une transfusion en post opératoire immédiat. Le retour du transit a eu lieu 48h après. L'antibiothérapie instaurée était Trixon-S (ceftriaxone 500mg et Sulbactam 250mg) 750mg deux fois par jour en IVD en attente des résultats de la pyoculture et antibiogramme. La pyoculture et antibiogramme a révélé la présence d'Enterobacter cloacae sensible à la norfloxacine. L'antibiothérapie a été réajustée à raison de Normet (5ml de suspension contient norfloxacine 100mg et metronidazol 100mg) 7,5ml 2 fois par jour pendant 7 jours. Les suites opératoires étaient bonnes.

## Discussion

L'adénite mésentérique suppurée est une pathologie très rarement rencontrée. Elle s'observe généralement chez les enfants entre 3 et 13 ans. Une étude faite en Afrique du sud a trouvé le streptocoque béta hémolytique et le staphylocoque doré à la culture du pus [1]. Une étude américaine a trouvé le peptostreptocoque et déclare que le diagnostic est plus simple à la laparotomie [2]. L'invagination intestinale est une urgence abdominale la plus fréquente de l'enfant. Dans 90 à 95% des cas elle est idiopathique. Malgré le fait qu'elle est idiopathique, on a trouvé une association avec une hypertrophie des ganglions mésentériques de cause infectieuse. Les germes couramment rencontré sont les virus: adénovirus, rotavirus, entérovirus, herpès virus humain 6 et 7, cytomégalovirus et le virus d'Epstein-Barr [3]. Mais le rotavirus est le plus souvent décrit [4]. Les bactéries associées à l'invagination intestinales sont les suivantes: Yersinia Enterocolitica, Staphylocoque Doré, Escherichia Coli O157; H7, Salmonella et Campylobacter [3]. De toutes ces bactéries, la plus fréquemment rencontrée est le Yersinia Enterocolitica [5–7]. Les parasites ont aussi été cités dans l'invagination intestinale. Ce sont: Entamoeba Histolitica, Trichuris Trichuira, Ascaris Lumbricoides, Ankylostoma et Giadia [3]. Une autre étude a révélé la présence d'Ascaris Lumbricoides [8]. Dans toutes ces littératures nous ne trouvons pas une invagination intestinale associée à une adénite mésentérique suppurée. La présence de l'Enterobacter Cloacae n'a pas été mentionnée dans les différentes littératures que nous avons mentionnées.

## Conclusion

L'adénite mésentérique suppurée est une pathologie rare de nos jours. Son diagnostic est difficile par les moyens d'imagerie dont nous disposons dans notre milieu. Son associations à une invagination intestinale est encore plus rare. Tout médecin qui prend en charge un enfant pour invagination intestinale aigue devra garder à l'esprit que l'association invagination intestinale aigue et adénite mésentérique suppurée est possible. Une ouverture de cet abcès dans la cavité abdominale peut se compliquer de péritonite qui complique le tableau d'invagination intestinale.
